# Botulinum toxin A for post-stroke spasticity: Insights from the French National Hospital Discharge Database (2015–2023)

**DOI:** 10.1093/esj/23969873251374771

**Published:** 2026-01-01

**Authors:** Djamel Bensmail, Anne Forestier, Jean-Yves Loze, Pierre Karam

**Affiliations:** Department of Physical and Rehabilitation Medicine, Raymond-Poincaré Teaching Hospital AP-HP, Université Paris-Saclay, Garches, France; Unité INSERM 1179, University of Versailles Saint-Quentin-En-Yvelines, Montigny-Le-Bretonneux, France; Ipsen, Boulogne-Billancourt, Île-de-France, France; Ipsen, Boulogne-Billancourt, Île-de-France, France; PKCS, Ecully, France

**Keywords:** Botulinum toxin, France, care pathway, spasticity, stroke

## Abstract

**Background:**

Botulinum neurotoxin type A (BoNT-A) is a well-established treatment for post-stroke spasticity. However, its real-world use remains underexplored. This study evaluated BoNT-A use trends among stroke survivors in France from 2015 to 2023.

**Methods:**

A retrospective cohort study was conducted using data from the French National Hospital Discharge Database. We analyzed stroke hospitalizations and BoNT-A treatment rates by age and care pathway. Among patients presenting with stroke between 2017 and 2019 who survived beyond 6 months post-stroke, we estimated the prevalence of patients with coded post-stroke spasticity, BoNT-A use, and time from stroke onset to spasticity coding and the first BoNT-A injection.

**Results:**

Between 2015 and 2023, 1,170,436 hospitalizations for stroke were recorded in France. BoNT-A treatment rates remained low, ranging from 1.4% in 2015 to 1.9% in 2022. BoNT-A treatment rates increased from 3.3% to 3.8% in stroke survivors aged 20–29 and from 1.0% to 1.6% in those aged 70–79 between 2015 and 2022. Patients who, during their care pathway, stayed in a neurovascular or neurorehabilitation unit were more likely to receive BoNT-A treatment—rising from 2.0% in 2015 to 2.6% in 2022 and 7.3% to 9.6%, respectively—than those managed in non-specialized units, where rates increased from 0.9% in 2015 to 1.1% in 2022. Among 287,370 patients presenting with stroke between 2017 and 2019, 37,692 (13.1%) were coded with post-stroke spasticity, 8056 (2.8%) received ⩾1 BoNT-A injection between 2017 and 2023, 4360 (1.5%) received ⩾3 injections, and 1003 (0.35%) received ⩾3 injections spaced ⩽6 months apart. The median time from stroke onset to spasticity coding was 96 days, and to the first BoNT-A injection 258 days.

**Conclusion:**

BoNT-A remains underutilized in the treatment of post-stroke spasticity in France. These results emphasize the need to enhance access to and adherence to BoNT-A therapy to optimize post-stroke spasticity management.

## Graphical Abstract

**Graphical abstract:**
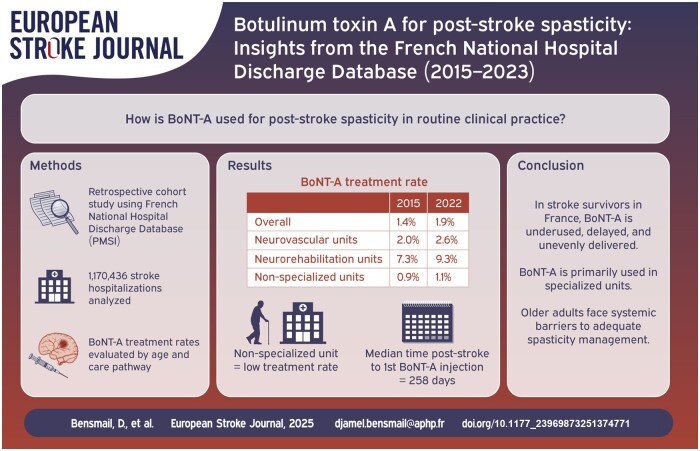

## Introduction

Botulinum neurotoxin type A (BoNT-A) has emerged as a cornerstone therapy for focal spasticity, recognized for its effectiveness and tolerability.^1–3^ Its increasing use over the years reflects its integral role in spasticity management within clinical practice.^4^ Indeed, BoNT-A has been used in a variety of conditions and symptoms, demonstrating its broad therapeutic potential.^1,4,5^ BoNT-A acts by blocking acetylcholine release at the neuromuscular junction, leading to reduced muscle overactivity and improved functional outcomes.^1,4^ In clinical practice, BoNT-A is administered for stroke with inter-injection intervals ranging from 12 to 16 weeks.^2,4^

Despite its inclusion in clinical guidelines and accumulating clinical evidence,^2,3^ prior analyses of real-world data from France (2014–2020) revealed underutilization of BoNT-A for post-stroke spasticity.^6^ Among 318,025 evaluated stroke survivors, only 10.7% were coded with post-stroke spasticity, 2.3% received at least one BoNT-A injection, and just 0.8% adhered to the recommended regimen of ⩾3 BoNT-A injections annually.^6^ The present study builds upon previous research by analyzing real-world records from the French National Hospital Discharge Database (*Programme de Médicalisation des Systèmes d’Information*, PMSI) from 2015 to 2023 to evaluate trends in BoNT-A use among stroke survivors across France. By examining updated data, we aimed to identify progress and persisting gaps in care pathways, focusing on access to and adherence to BoNT-A therapy.

## Methods

### Study design and data source

This nationwide, population-based, retrospective cohort study utilized data from the French PMSI database covering the period from 2015 to 2023. This exhaustive medico-administrative database contained standardized hospital records across France, including diagnoses and medical procedures. Diagnoses were coded according to the International Classification of Diseases 10th Edition (ICD-10), categorized as the primary diagnosis (PD; the reason behind the patient’s hospitalization), related diagnoses (RD; all conditions potentially related to the PD), and significantly associated diagnoses (SAD; all complications and morbidities that could influence the course of hospitalization). Medical procedures were coded according to the French Joint Classification of Medical Procedures (*Classification Commune des Actes Médicaux*, CCAM). The present project encompassed several analyses, three of which have been published.^6–8^

This study was performed in accordance with French laws and regulations and the Declaration of Helsinki. Access to the PMSI data was granted by the French Data Protection Agency (*Commission Nationale de l’Informatique et des Libertés*, CNIL; CNIL MR006). This study did not involve direct participation of humans, and all extracted data were anonymized.

### Study population

The study included adult and pediatric stroke survivors identified using ICD-10 codes for stroke (I61, I62, I63, I64) between 2015 and 2023. Patients were classified by age and by admission to specialized neurovascular units (NVU) or neurovascular intensive care units (NVICU). They were further categorized based on their patient care pathway within 2 months following discharge from critical care, distinguishing between neuro-rehabilitation units (NRU) managed by physical medicine and rehabilitation specialists, non-neuro-rehabilitation units (Non-NRU) managed by multidisciplinary specialists, and no rehabilitation.

Stroke survivors who received at least one BoNT-A injection into striated muscles during the study period were identified using the CCAM codes “PCLB002” (injections without electromyographic guidance) and “PCLB003” (injections with guidance). Each recorded BoNT-A injection was systematically linked to stroke as the underlying etiology using a tailored algorithm. This algorithm assessed diagnostic codes (PD, RD, and SAD) recorded during the injection-related hospital stay, as well as any other hospital stays between 2015 and 2023. In cases where multiple etiologies were identified for the same patient, the most frequently coded etiology was retained for analysis. This ensured that each patient was assigned a single, most relevant condition justifying BoNT-A administration. As BoNT-A treatment for stroke survivors is administered in hospital settings in France, BoNT-A injections were identified during stays in private and public hospitals (including day hospital visits), and during ambulatory visits in public hospitals only.

### Outcomes

We assessed the total number of stroke hospitalizations in France between 2015 and 2023. We evaluated the BoNT-A treatment rate from 2015 to 2022, by examining the proportion of stroke survivors who received at least one BoNT-A injection each year. BoNT-A treatment persistence, evaluated from 2015 to 2021, was defined as the proportion of BoNT-A-treated patients who received at least three BoNT-A injections within 2 years following their stroke. Among patients presenting with stroke between 2017 and 2019 who survived beyond 6 months post-stroke, we further analyzed the prevalence of post-stroke spasticity coding, the extent of BoNT-A use (at least one or three injections), and the time from stroke onset to the development of spasticity, and to the first BoNT-A injection.

### Statistical analysis

Descriptive analyses were performed using numbers and percentages for categorical variables, and mean, standard deviation (SD), median, and interquartile range (IQR) for continuous variables. Missing data was not imputed, and all coding was discontinued in cases of patient death. All analyses were performed using SQL Server software (version 2022; Microsoft, Redmond, WA, USA).

## Results

### Stroke hospitalizations in France

**Figure 1.**
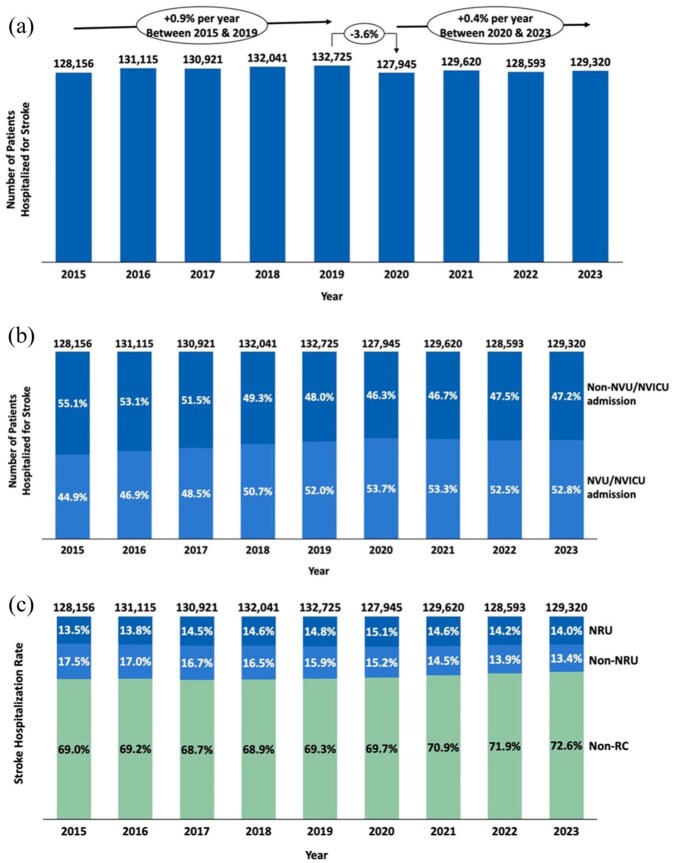
Overview of stroke hospitalizations in France from 2015 to 2023. (a) Distribution of hospitalizations in specialized neurovascular units (NVU) or neurovascular intensive care units (NVICU; b), and hospitalization in neuro-rehabilitation care (NRU), non-neuro-rehabilitation care (Non-NRU), and non-Rehabilitation care (Non-RC; c).

Between 2015 and 2023, a total of 1,170,436 hospitalizations for stroke were recorded in France. Except during the COVID-19 pandemic (2019–2020), stroke-related hospitalizations slightly increased from 128,156 in 2015–129,320 in 2023, reflecting a stable trend with an annual increase of less than 1% (Figure 1(a)). Over this period, the types of units where patients were hospitalized evolved. In 2015, 44.9% of stroke hospitalizations took place in neurovascular units (NVU) or neurovascular intensive care units (NVICU), compared to 52.8% in 2023 (Figure 1(b)). However, the distribution of stroke hospitalizations across rehabilitation care categories remained relatively stable during the same period. In 2015, 17.5% of stroke hospitalizations occurred in non-neuro-rehabilitation units (Non-NRU), 13.5% in neuro-rehabilitation units (NRU), and 69.0% did not receive rehabilitation care. Likewise, in 2023, stroke hospitalization rates were 13.4% in Non-NRU, 14.0% in NRU, and 72.6% did not receive rehabilitation care (Figure 1(c)).

### BoNT-A treatment rates and trends

**Figure 2.**
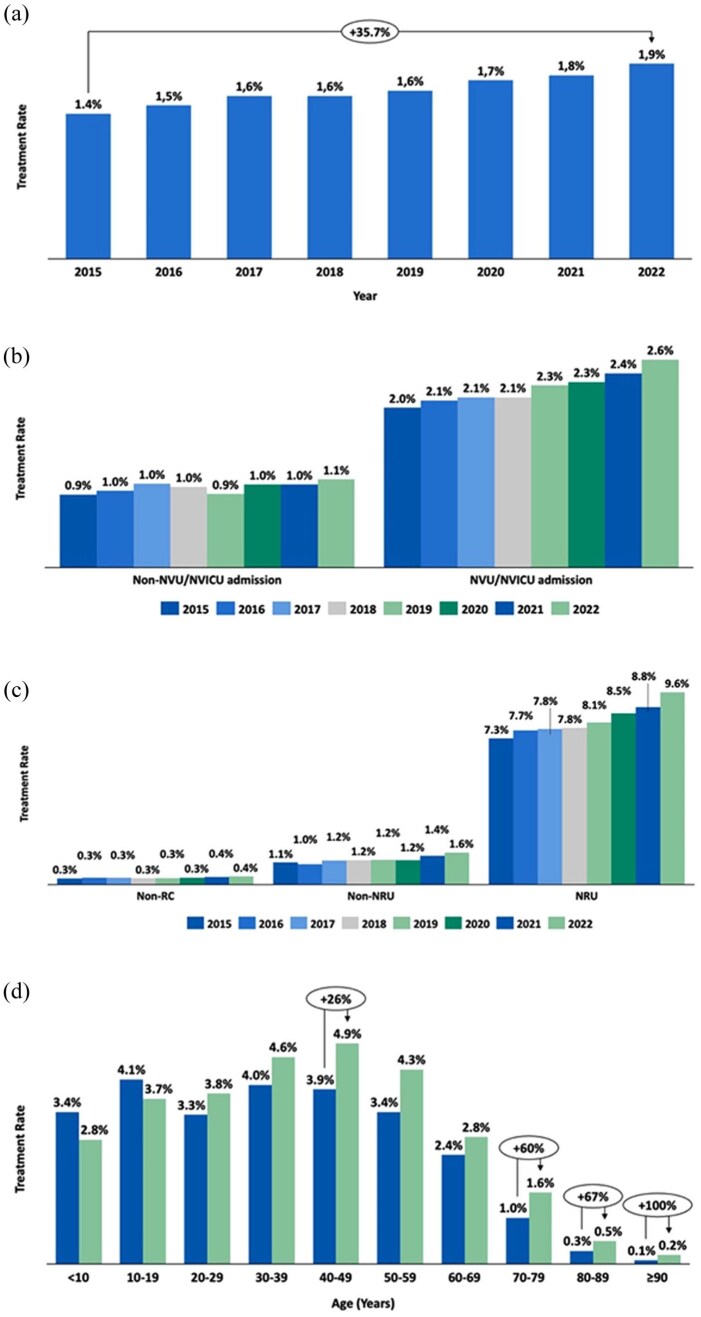
Botulinum neurotoxin type A use among stroke survivors in France. (a) Overall treatment rate from 2015 to 2022; (b) Treatment rate by admission to neurovascular units (NVU) and neurovascular intensive care units (NVICU); (c) Treatment rate by neuro-rehabilitation care (NRU), non-neuro-rehabilitation care (Non-NRU), and non-Rehabilitation care (Non-RC); (d) Treatment rate by age group between 2015 and 2022.

**Figure 3.**
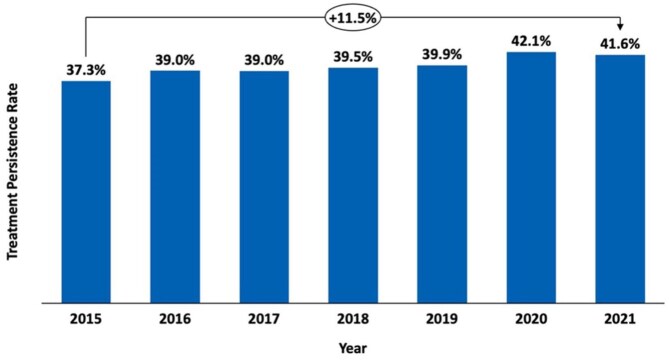
Botulinum neurotoxin type A (BoNT-A) treatment persistence, defined as the proportion of BoNT-A-treated patients receiving at least three injections within 2 years post-stroke.

The overall BoNT-A treatment rate among stroke patients in France rose from 1.4% in 2015–1.9% in 2022, marking a 35.7% increase (Figure 2(a); Supplemental Table 1). BoNT-A treatment rate was higher in patients staying in specialized neurovascular units (NVU/NVICU), increasing from 2.0% in 2015 to 2.6% in 2022. By contrast, in non-specialized units, the treatment rate increased from 0.9% to 1.1% over the same period (Figure 2(b); Supplemental Table 2). In patients receiving rehabilitation care, the BoNT-A treatment rate was consistently higher in neuro-rehabilitation units (NRU), rising from 7.3% in 2015 to 9.6% in 2022. By comparison, BoNT-A treatment rate in non-neuro-rehabilitation units (Non-NRU) increased from 1.1% to 1.6%, while in patients who did not receive rehabilitation care, it increased from 0.3% to 0.4%, respectively (Figure 2(c); Supplemental Table 3). Among stroke survivors aged 20 years and older, the BoNT-A treatment rate consistently increased across all age categories between 2015 and 2022 (Figure 2(d); Supplemental Table 4). BoNT-A treatment persistence, defined as the proportion of BoNT-A-treated patients receiving at least three injections within 2 years post-stroke, increased from 37.3% in 2015 to 41.6% in 2021 (Figure 3).

### BoNT-A use in the 2017–2019 cohort of patients with stroke

**Figure 4.**
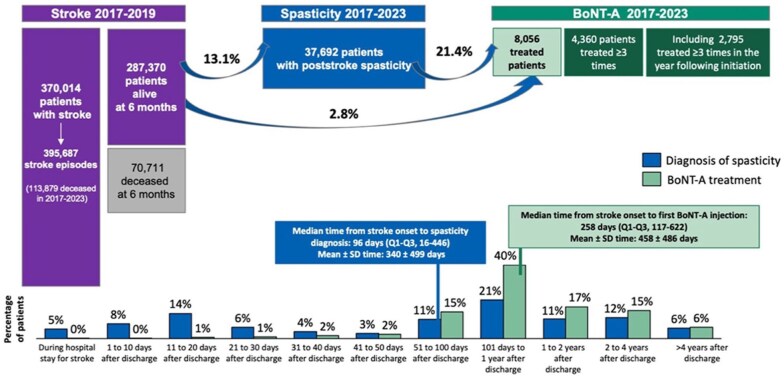
Care pathway of patients admitted for acute stroke between 2017 and 2019 in hospitals across France who were followed-up until 2023. Of the 370,014 patients with stroke, 2130 were coded with spasticity or received BoNT-A prior to the stroke event. Additionally, 9803 patients had a stroke in 2015–2016. These patients were hence excluded from the analysis, along with 70,711 patients who died within 6 months post-stroke. BoNT-A: botulinum neurotoxin type A; Q: quartile; SD: standard deviation.

Between 2017 and 2019, a total of 370,014 patients (both adult and pediatric) experienced at least one stroke event (395,687 events in total), 70,711 of whom died within the first 6 months, leaving 287,370 patients to be followed up until 2023. Of these 287,370 stroke survivors, 37,692 (13.1%) were coded with post-stroke spasticity, 8056 (2.8%) received at least one BoNT-A injection between 2017 and 2023, and 4360 (1.5%) received at least three injections. The median (IQR) time from stroke onset to spasticity coding was 96 (16–446) days. Overall, 21.4% of patients coded with post-stroke spasticity (8056 out of 37,692) received at least one BoNT-A injection, with the median (IQR) time between stroke onset and the first BoNT-A injection of 258 (117–622) days (Figure 4).

**Figure 5.**
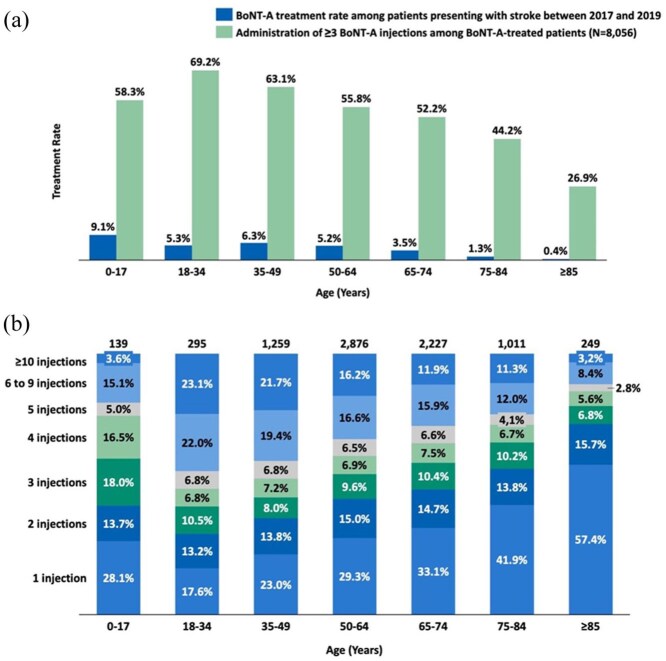
Botulinum neurotoxin type A (BoNT-A) treatment rate by age among patients admitted for acute stroke between 2017 and 2019 (a) and frequency of BoNT-A injections among 8056 BoNT-A-treated patients with stroke (b).

**Figure 6.**
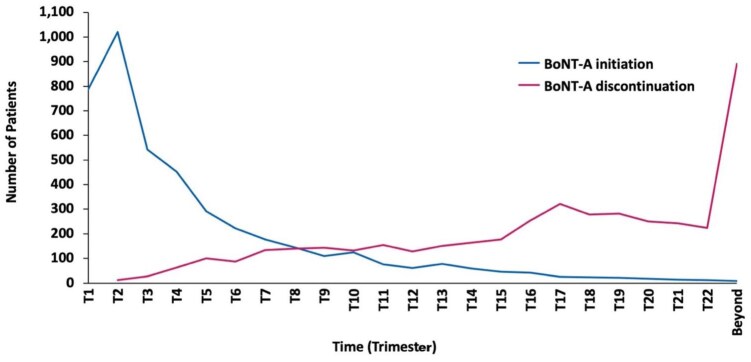
Kinetics of initiation and discontinuation of botulinum neurotoxin type A (BoNT-A) therapy among 4360 patients with stroke treated with ⩾3 BoNT-A injections.

An age-specific analysis indicated a steady decrease in the proportion of patients with stroke treated with at least one BoNT-A injection, from 9.1% in patients aged ⩽ 17 years to 0.4% in those aged ⩾ 85 years (Figure 5(a); Supplemental Table 5). Among 8,056 BoNT-A-treated patients with stroke, 57.4% of those aged ⩾ 85 years received only one BoNT-A injection, compared to 17.6% of patients aged 18–34 years (Figure 5(b); Supplemental Table 6). For the 4360 patients treated with at least three BoNT-A injections, starting from the 10th trimester, the number of patients who discontinued BoNT-A therapy surpassed the number of those who initiated it (Figure 6).

Among the 287,370 patients presenting with stroke between 2017 and 2019, 1003 patients (0.35%) received at least three BoNT-A injections without an interruption exceeding 6 months, in accordance with recommendations suggesting a treatment interval of 3–4 months.^2^ These patients remained under BoNT-A treatment at the end of 2023. The median intervals between BoNT-A re-injections ranged from 106 days (3.5 months) to 136 days (4.5 months) across 15 re-injection events. The mean intervals varied from 3.7 to 6.9 months, with the longest mean interval observed at the first re-injection (210 days) and the shortest at the final BoNT-A re-injection (113 days; Table 1) .

**Table 1.** Botulinum neurotoxin type A (BoNT-A) re-injection intervals (in days) for patients presenting with stroke between 2017 and 2019.

BoNT-A re-injection	Median interval (range)	Mean ± standard deviation interval (days)
First re-injection	134 (104–217)	210 ± 226
2	136 (105–202)	198 ± 197
3	135 (110–194)	188 ± 176
4	133 (106–182)	174 ± 142
5	133 (105–182)	168 ± 131
6	130 (105–176)	161 ± 116
7	126 (102–168)	152 ± 98
8	126 (101–161)	146 ± 92
9	120 (99–154)	136 ± 67
10	121 (100–154)	135 ± 62
11	119 (98–140)	129 ± 60
12	113 (98–133)	125 ± 61
13	112 (98–133)	123 ± 65
14	106 (95–126)	116 ± 53
15	111 (92–126)	113 ± 44

## Discussion

Our analysis of the comprehensive French PMSI database provided valuable insights into the utilization patterns of BoNT-A for managing spasticity among stroke survivors in France between 2015 and 2023. Although accumulating evidence supports the efficacy of BoNT-A in reducing spasticity and improving functional outcomes,^1–3^ our findings indicate suboptimal BoNT-A use in post-stroke care. Indeed, despite stable hospitalization rates for stroke, the BoNT-A treatment rate among stroke survivors was low, ranging from 1.4% in 2015 to 1.9% in 2022. These findings underscore a remaining gap in the long-term management of post-stroke spasticity and point to barriers in both access to and adherence to BoNT-A treatment. Potential contributing factors include limited availability of trained specialists to administer BoNT-A, regional disparities in access to multidisciplinary rehabilitation services, and logistical or administrative constraints such as appointment delays or insufficient awareness about spasticity among referring physicians.^9–11^

To improve adherence to BoNT-A therapy, an integrated approach is essential. Our data indicated that patients staying in specialized neurovascular units (NVU/NVICU) were more likely to receive BoNT-A treatment compared to those in non-specialized units. The structured environment of NVUs and NVICUs, which involves neurologists, rehabilitation specialists, and multidisciplinary teams, likely contributed to the improved recognition of spasticity and an increased likelihood of BoNT-A treatment.^9^ However, access to specialist spasticity services remains challenging, as many healthcare units are not adequately resourced to address the needs of stroke survivors with spasticity.^10^ A retrospective, observational study from Canada evaluating BoNT-A use for focal spasticity found that the median time from hospital admission to the first physical medicine and rehabilitation physician or neurologist claim was 2.8 years.^11^ This highlights the delays in accessing specialist care, which may lead to suboptimal outcomes for stroke survivors who could benefit from earlier interventions.

Neurorehabilitation is also a critical component of patient care and recovery.^4,10^ In the present study, BoNT-A treatment rates were consistently higher among stroke survivors who received care in neuro-rehabilitation units (NRU) compared to those in non-neuro-rehabilitation units (Non-NRU) or those who did not receive rehabilitation. This underscores the pivotal role of dedicated neuro-rehabilitation programs in fostering long-term adherence to BoNT-A therapy. It also emphasizes the importance of patient care pathways, where even if BoNT-A injections are not administered during critical care, a patient’s stay in critical care may leads to a transfer to other specialized rehabilitation units, where they are more likely to receive BoNT-A treatment. In addition to neurorehabilitation, various adjuvant treatments have the potential to improve therapeutic outcomes following BoNT-A injections. These include adhesive taping, casting, electrical stimulation, modified constraint-induced movement therapy, physiotherapy, and splinting. Such interventions complement the effects of BoNT-A by improving muscle function and further reducing spasticity.^4^ Ultimately, a well-structured care pathway that integrates neuro-rehabilitation and supportive therapies can increase the likelihood of BoNT-A use and improve overall patient recovery.

Age was another key determinant of BoNT-A use, with older patients being less likely to receive BoNT-A therapy, as observed in our study, and consistent with other research.^12^ This may be due to multiple factors, including lower referral rates, greater comorbidity burden, increased mortality, or clinical hesitancy in initiating therapy.^13^ Furthermore, in France, the education of geriatricians regarding the technical aspects of BoNT-A injection is limited, which may contribute to this disparity. Ensuring equitable access to BoNT-A therapy across all age groups requires increased awareness among healthcare providers and standardized referral processes that prioritize functional outcomes regardless of patient age.^10^

Our study found a median of 258 days between stroke onset and the first BoNT-A injection, which is shorter than the median time of 364 days reported for Spanish patients treated with BoNT-A for post-stroke spasticity.^12^ It is also shorter than the median time of 2.9 years for receiving the first BoNT-A injection for focal spasticity in Canadian patients.^11^ Initiating BoNT-A treatment as early as possible is paramount to maximize its effectiveness and achieve a greater reduction in muscle tone.^4,14^

The analysis of re-injection intervals for BoNT-A among stroke survivors from 2017 to 2019 revealed median intervals ranging from 3.5 to 4.5 months. This aligns with the clinical recommendation for BoNT-A re-injections every 3–4 months to achieve a stable plateau of improvement.^2,15^ However, adherence to BoNT-A treatment regimens was suboptimal in the cohort of 287,370 patients admitted for acute stroke between 2017 and 2019 who were followed until 2023. Of these, only 1.5% received at least three BoNT-A injections during the observation period. Moreover, only 0.35% of patients received at least three BoNT-A injections within a time interval of up to 6 months. This reflects a persisting gap between clinical guidelines and real-world practice, emphasizing the need for improved adherence to treatment recommendations.

A cross-sectional study from Italy, conducted during the COVID-19 pandemic in patients with spasticity after stroke and traumatic brain injury treated with BoNT-A, found that discontinuation of BoNT-A was associated with worsening of perceived spasticity and a loss of independence.^16^ In addition, a pharmacoeconomic study conducted in Australia demonstrated that continuing BoNT-A treatments beyond the fourth cycle is a cost-effective strategy.^17^ These findings emphasize the importance of adherence to BoNT-A treatment regimens, not only for achieving optimal clinical outcomes but also for maintaining patient independence and minimizing long-term healthcare costs.^16,17^

The present study has limitations inherent to the retrospective analysis of electronic health records (the PMSI database using ICD-10 and CCAM codes). These limitations include the potential inaccuracies in diagnostic coding and missing or incomplete clinical information. Of note, current ICD-10 coding does not differentiate between focal and generalized spasticity, limiting the accuracy of symptom tracking. Another limitation of the PMSI database is that it does not contain data from ambulatory visits in private hospitals. BoNT-A injections were coded during private and public hospitals stays, including day hospital visits and during ambulatory visits in public hospitals only. Since ambulatory activity is minimal in the private sector for post-stroke spasticity management, primarily due to cost-effectiveness and reimbursement issues,^6^ this limitation is unlikely to significantly affect estimates of BoNT-A treatment rates. Finally, due to the descriptive nature of this study, outcomes related to the effectiveness or safety of BoNT-A were not evaluated.

Despite these limitations, our study was strengthened by the use of the PMSI national database, which covers the entire population of France (over 66 million people), thereby minimizing the risk of selection bias. The study also benefited from exhaustive data collection on BoNT-A use, with an extended follow-up period from 2015 until 2023. Furthermore, the identification of stroke, post-stroke spasticity, and BoNT-A injections relied on ICD-10 diagnostic codes and CCAM procedure codes within the PMSI database. The use of multiple diagnostic codes (PD, RD, and SAD) and a tailored algorithm to link BoNT-A injections to stroke events enhanced the accuracy of case identification.

## Conclusions

In a contemporary French population of stroke survivors, BoNT-A therapy for spasticity remains suboptimal and poorly adhered to. The proportion of stroke survivors treated with BoNT-A ranged from 1.4% in 2015 to 1.9% in 2022. These findings underscore the need to enhance access to and adherence to BoNT-A therapy to optimize the management of post-stroke spasticity.

## Supplementary Material

sj-docx-1-eso_23969873251374771
