# Supplementary material for: Botulinum toxin A for post-stroke spasticity: Insights from the French National Hospital Discharge Database (2015–2023)
Source: Eur Stroke J. 2026 Jan 1;11(1):23969873251374771. doi: 10.1093/esj/23969873251374771 (PMC12866233; doi:10.1093/esj/23969873251374771)
Supplement: sj-docx-1-eso_23969873251374771 [file sj-docx-1-eso_23969873251374771.docx]

**Supplementary Table 1.** Number of patients with stroke treated with botulinum neurotoxin type A (BoNT-A) over the study period

| **Patients with stroke** | **Year** | | | | | | | |
| --- | --- | --- | --- | --- | --- | --- | --- | --- |
|  | **2015** | **2016** | **2017** | **2018** | **2019** | **2020** | **2021** | **2022** |
| Treated | 1,779 | 1,933 | 2,041 | 2,060 | 2,138 | 2,185 | 2,288 | 2,413 |
| Total patients with stroke | 128,156 | 131,115 | 130,921 | 132,041 | 132,725 | 127,945 | 129,620 | 128,593 |

**Supplementary Table 2.** Number of patients treated with botulinum neurotoxin type A (BoNT-A) by neurovascular units (NVU) and neurovascular intensive care units (NVICU), over the study period

| **Patients with stroke** | **Year** | | | | | | | |
| --- | --- | --- | --- | --- | --- | --- | --- | --- |
|  | **2015** | **2016** | **2017** | **2018** | **2019** | **2020** | **2021** | **2022** |
| Non-NVU | 70,608 | 69,613 | 67,410 | 65,061 | 63,756 | 59,188 | 60,527 | 61,024 |
| Not treated | 69,969 | 68,954 | 66,708 | 64,413 | 63,175 | 58,581 | 59,905 | 60,357 |
| Treated | 639 | 659 | 702 | 648 | 581 | 607 | 622 | 667 |
| NVU/NVICU | 57,548 | 61,502 | 63,511 | 66,980 | 68,969 | 68,757 | 69,093 | 67,569 |
| Not treated | 56,408 | 60,228 | 62,172 | 65,568 | 67,412 | 67,179 | 67,427 | 65,823 |
| Treated | 1,140 | 1,274 | 1,339 | 1,412 | 1,557 | 1,578 | 1,666 | 1,746 |

**Supplementary Table 3.** Number of patients treated with botulinum neurotoxin type A (BoNT-A) by neuro-rehabilitation units (NRU), non-neuro-rehabilitation units (Non-NRU), and non-rehabilitation care, over the study period

| **Patients with stroke** | **Year** | | | | | | | |
| --- | --- | --- | --- | --- | --- | --- | --- | --- |
|  | **2015** | **2016** | **2017** | **2018** | **2019** | **2020** | **2021** | **2022** |
| Non-NRU | 22,413 | 22,231 | 21,882 | 21,846 | 21,105 | 19,391 | 18,763 | 17,874 |
| Not treated | 22,166 | 22,005 | 21,622 | 21,581 | 20,845 | 19,155 | 18,494 | 17,588 |
| Treated | 247 | 226 | 260 | 265 | 260 | 236 | 269 | 286 |
| NRU | 17,312 | 18,137 | 19,033 | 19,256 | 19,621 | 19,325 | 18,923 | 18,240 |
| Not treated | 16,050 | 16,742 | 17,557 | 17,753 | 18,034 | 17,676 | 17,251 | 16,489 |
| Treated | 1,262 | 1,395 | 1,476 | 1,503 | 1,587 | 1,649 | 1,672 | 1,751 |
| Non-rehabilitation care | 88,431 | 90,747 | 90,006 | 90,939 | 91,999 | 89,229 | 91,934 | 92,479 |
| Not treated | 88,161 | 90,435 | 89,701 | 90,647 | 91,708 | 88,929 | 91,587 | 92,103 |
| Treated | 270 | 312 | 305 | 292 | 291 | 300 | 347 | 376 |

**Supplementary Table 4.** Number of patients by age group and by botulinum neurotoxin type A (BoNT-A) treatment status (in 2015 and 2022)

| **Timepoint** | **Treatment status** | **Age group (years)** | | | | | | | | |
| --- | --- | --- | --- | --- | --- | --- | --- | --- | --- | --- |
|  |  | **<10** | **10-19** | **20-29** | **30-39** | **40-49** | **50-59** | **60-69** | **70-79** | **80-89** |
| 2015 | Not treated | 402 | 352 | 994 | 2,299 | 6,255 | 12,161 | 21,244 | 27,722 | 14,497 |
|  | Treated | 14 | 15 | 34 | 95 | 252 | 424 | 526 | 288 | 12 |
| 2022 | Not treated | 353 | 342 | 993 | 2,286 | 5,853 | 12,678 | 21,670 | 33,046 | 14,663 |
|  | Treated | 9 | 13 | 39 | 110 | 301 | 571 | 630 | 534 | 30 |

**Supplementary Table 5.** Botulinum neurotoxin type A (BoNT-A) treatment rates and persistence by age group among 287,370 patients presenting with stroke between 2017 and 2019

| **Patients with stroke between 2017 and 2019** | **Age group (years)** | | | | | | |
| --- | --- | --- | --- | --- | --- | --- | --- |
|  | **0-17** | **18-34** | **35-49** | **50-64** | **65-74** | **75-84** | **≥85** |
| Total number of patients with stroke | 1,529 | 5,542 | 19,857 | 55,350 | 64,103 | 75,209 | 65,780 |
| Treated at least once | 139 | 295 | 1,259 | 2,876 | 2,227 | 1,011 | 249 |
| Treatment rate | 9.1% | 5.3% | 6.3% | 5.2% | 3.5% | 1.3% | 0.4% |
| Treated at least 3 times | 81 | 204 | 795 | 1,604 | 1,162 | 447 | 67 |
| Persistence rate | 58.3% | 69.2% | 63.1% | 55.8% | 52.2% | 44.2% | 26.9% |

**Supplementary Table 6.** Number of patients per age group and per number of injections among 8,056 BoNT-A-treated patients with stroke between 2017 and 2019

| **Number of injections** | **Age group (years)** | | | | | | |
| --- | --- | --- | --- | --- | --- | --- | --- |
|  | **0-17** | **18-34** | **35-49** | **50-64** | **65-74** | **75-84** | **≥85** |
| ≥10 | 5 | 68 | 273 | 465 | 264 | 114 | 8 |
| 6 to 9 | 21 | 65 | 244 | 478 | 353 | 121 | 21 |
| 5 | 7 | 20 | 86 | 186 | 148 | 41 | 7 |
| 4 | 23 | 20 | 91 | 198 | 166 | 68 | 14 |
| 3 | 25 | 31 | 101 | 277 | 231 | 103 | 17 |
| 2 | 19 | 39 | 174 | 430 | 327 | 140 | 39 |
| 1 | 39 | 52 | 290 | 842 | 738 | 424 | 143 |
